# Simultaneously controlling heat conduction and infrared absorption with a textured dielectric film to enhance the performance of thermopiles

**DOI:** 10.1038/s41378-021-00264-z

**Published:** 2021-05-11

**Authors:** Yunqian He, Yuelin Wang, Tie Li

**Affiliations:** 1 Science and Technology on Microsystem Laboratory, Shanghai Institute of Microsystem and Information Technology, Chinese Academy of Sciences, 200050 Shanghai, China; 2 University of Chinese Academy of Sciences, 100049 Beijing, China

**Keywords:** Engineering, Physics

## Abstract

The heat conduction and infrared absorption properties of the dielectric film have a great influence on the thermopile performance. Thinning the dielectric film, reducing its contact area with the silicon substrate, or adding high-absorptivity nanomaterials has been proven to be effective in improving thermopiles. However, these methods may result in a decrease in the structural mechanical strength and increases in the fabrication complexity and cost. In this work, a new performance-enhancement strategy for thermopiles by simultaneously controlling the heat conduction and infrared absorption with a TExtured DIelectric (TEDI) film is developed and presented. The TEDI film is formed in situ by a simple hard-molding process that is compatible with the fabrication of traditional thermopiles. Compared to the control FLat DIelectric (FLDI) film, the intrinsic thermal conductance of the TEDI film can be reduced by ~18–30%, while the infrared absorption can be increased by ~7–13%. Correspondingly, the responsivity and detectivity of the fabricated TEDI film-based thermopile can be significantly enhanced by ~38–64%. An optimized TEDI film-based thermopile has achieved a responsivity of 156.89 V·W^−1^ and a detectivity of 2.16 × 10^8^ cm·Hz^1/2^·W^−1^, while the response time constant can remain <12 ms. These results exhibit the great potential of using this strategy to develop high-performance thermopiles and enhance other sensors with heat transfer and/or infrared absorption mechanisms.

## Introduction

Cost-effective thermopiles have been widely used as sensing elements for noncontact infrared thermometers^1,2^, uncooled infrared imagers^3,4^, nondispersive infrared (NDIR) sensing systems^5,6^, thermoelectric generators^7,8^, and gas/heat flow sensors^9,10^. Enhancing the thermopile can facilitate the development of high-performance temperature measurement, target tracking, infrared detection, and thermoelectric power in industrial and civil applications. Generally, a cost-effective thermopile is composed of a thermocouple array and a complementary metal–oxide–semiconductor (CMOS)-compatible dielectric film infrared absorber. The infrared absorber can absorb infrared photon energy and convert it into thermal energy. Then, the carriers in the thermocouple move from the hot to the cold junction, which can accumulate to form a potential difference under thermal energy. According to both infrared (IR)-thermal and thermal-electric conversions^11,12^, the high performance of the thermopile is mainly dependent on the sufficient thermocouple number, large Seebeck coefficient difference, low heat conductance, and high infrared absorption. With the development of MEMS technology, the number of thermocouples increased from a few pairs to more than one hundred^13,14^, while the duty factor of the thermocouple array was close to ~90%^15,16^. Then, the Seebeck coefficient difference of the typical poly-Si/metal, poly-Si/poly-Si, and single-crystalline (SC)-Si/metal thermocouples reached more than 150 μV·K^−1^, 250 μV·K^−1^, and 450 μV·K^−1^, respectively, by adjusting the implantation concentration in these materials^17–20^. Although the Seebeck coefficient difference of the last two is higher than that of poly-Si/metal, for the poly-Si/metal thermocouple, it is easy to integrate more thermocouples.

Unfortunately, large heat conduction can be caused by the high thermal conductivity of the metal and silicon substrate^21^. Some thermopile structures, such as suspended films^22,23^ and cantilever beams^24,25^, have been designed to lower the heat conduction by reducing the contact area between the infrared absorber and the silicon substrate. Moreover, a CMOS-compatible SiO_2_ or SiN_x_ dielectric film is usually used as the infrared absorber due to its low thermal conductivity^26,27^. The thermal conductivity of SiO_2_ and SiN_x_ films is as low as 1.4 W·m^−1^·K^−1^ and 20 W·m^−1^·K^−1^, respectively. Furthermore, some microporous films and micro/nanomaterials have shown excellent thermal insulation properties (<1 m^−1^·K^−1^), which have been demonstrated by various test methods^28–31^. These materials may have great potential in achieving ultralow thermal conductivity when their process compatibility issue can be resolved. In addition to the above factors, the performance of the thermopile is also limited by the relatively low infrared absorption (~0.7–0.8 within 8–14 µm) of the SiO_2_ or SiN_x_ dielectric film^32,33^. There may be a great prospective opportunity to enhance thermopiles by improving the infrared absorption of the FLDI film-based infrared absorber. Coating high-absorptivity black nanomaterials (e.g., metal black^14^, SU-8 photoresist^34^, and carbon black^35^) onto the surface of the FLDI film as the infrared absorber has become an effective method to obtain high infrared absorption. The infrared absorption of these black nanomaterials can reach >0.9 based on the multiple reflection absorption of incident light^36–39^. Similarly, a black microtip forest formed by etching the dielectric film can also be used to enhance infrared absorption^40,41^. In addition, some advanced metamaterials have been introduced as the infrared absorber to achieve infrared selective absorption owing to the electromagnetic resonance at some specific wavelengths^42–44^. A thermopile with a metal-insulator-metal (MIM) plasmonic metamaterial absorber has achieved wavelength-selective or polarization-selective absorption by adjusting the material, shape, size, or thickness of the MIM structure^45–48^. Furthermore, advanced bioinspired engineering may also offer a promising alternative approach in accelerating the development of ultrahigh sensitivity infrared detection by using bioinspired infrared sensing materials and systems^49^. Currently, thinning the dielectric film, reducing the contact area, forming a microporous film, or using micro/nanomaterials can lower the thermal conductance of the infrared absorber, while adding high-absorptivity black nanomaterials or absorption-selective advanced metamaterials can compensate for the infrared absorption. However, these methods may result in a decrease in the structural mechanical strength and increases in the complexity, cost, and CMOS incompatibility of thermopile fabrication.

The use of a TEDI film has been considered to be a simple and effective method to overcome these disadvantages by equivalent heat conduction extension and multiple reflection absorption of incident light in our previous works^50,51^. Here, control via the TEDI film of the heat conduction and infrared absorption as well as performance enhancement of the TEDI film-based thermopile are systematically analyzed and demonstrated. We develop and present a new performance-enhancement strategy, simultaneously controlling the heat conduction and infrared absorption with a TEDI film, to enhance thermopiles and other sensors with heat transfer or infrared absorption mechanisms.

## Materials and methods

### Thermopile basics

**Fig. 1:**
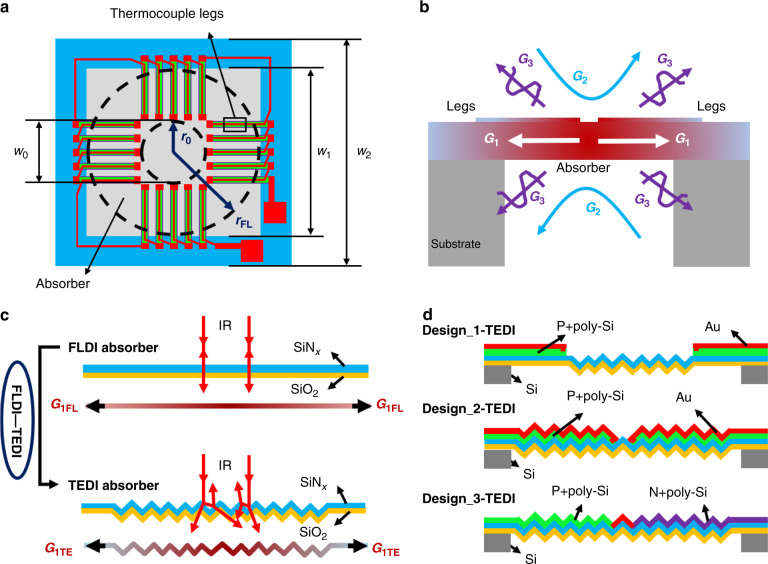
Characteristics of the FLDI film-based and TEDI film-based thermopiles. **a**, **b** Top view and heat transfer (thermal conductance of the structure *G*_1_, convection *G*_2_, and radiation *G*_3_) of the traditional FLDI film-based thermopile, **c** characteristic comparison between the FLDI film and the designed TEDI film, and **d** cross-sections of the designed TEDI film-based thermopiles.

Figure 1a shows a traditional closed membrane-type MEMS thermopile with a poly-Si/metal thermocouple array. The infrared absorber is capable of absorbing infrared photonic energy and converting it into thermal energy to heat the thermoelectric material and form a potential difference. The output voltage response *V* of the thermopile can be derived from the series potential of all thermocouples and expressed as:^11^

1 $$V = N{\Delta}\alpha _{12}\phi _0A_0\frac{\eta }{G}$$

where *N* is the number of thermocouples, Δ*α*_12_ is the Seebeck coefficient difference between the two thermocouple materials, *ϕ*_0_ is the infrared radiation power density, *A*_0_ is the absorber area, *η* is the infrared absorptivity, and *G* is the thermal conductance.

The heat transfer of the FLDI film-based thermopile is shown in Fig. 1b. The thermal conductance *G* is mainly composed of the thermal conductance of the structure *G*_*1*_, convection *G*_*2*_, and radiation *G*_*3*_. Since the thermopile is usually packaged in a shell and works in the air with a low-convection coefficient and the temperature difference between the thermopile and the environment is usually very small, it can be assumed that the thermal conduction loss through convection and radiation is negligible and that all heat is transferred laterally through the thermocouple and the FLDI film to the silicon substrate. Thus, the thermal conductance *G* of the thermopile can be simplified as^11^:

2 $$G = G_{1{\mathrm{leg}}} + G_{1{\mathrm{FL}}} = N\mathop {\sum}\limits_i {\frac{{\lambda _ia_i}}{{r_{{\mathrm{FL}}} - r_0}} + \mathop {\sum}\limits_j {\frac{{2\pi \lambda _jt_j}}{{\ln (r_{{\mathrm{FL}}}/r_0)}}} }$$

where *λ*_*i*_ and *λ*_*j*_ are the thermal conductivities of the *i*th thermoelectric material and the *j*th dielectric film, *a*_*i*_ is the cross-sectional area of the *i*th thermocouple leg, *t*_*j*_ is the thickness of the *j*th dielectric film, and *r*_FL_ and *r*_0_ are the distances of the heat conduction to the substrate and from the center to the hot junction of the thermocouple.

The responsivity *Rv*, detectivity D*, and response time constant *τ* are the main figures of merit for thermopiles. The responsivity, which is similar to the sensitivity of sensors, is calculated by normalization to the radiation power density and the absorber area. The detectivity further takes into account the noise voltage of the thermocouple resistance, which can allow the comparison of thermopiles with different structures. These performances can be expressed as^11^:

3 $$Rv = \frac{V}{{A_0\phi _0}}{\mathrm{ = }}N{\Delta}\alpha _{12}\frac{\eta }{G}$$

4 $$D^\ast = Rv\sqrt {\frac{{A_0{\Delta}f}}{{4kTR}}} = N{\Delta}\alpha _{12}\sqrt {\frac{{A_0{\Delta}f}}{{4kTR}}} \frac{\eta }{G}$$

5 $$\tau {\mathrm{ = }}\mathop {\sum}\limits_i {v_i\rho _iH_i} /G$$

where Δ*f* is the noise bandwidth (generally 1 Hz), *k* is the Boltzmann constant, *T* is the ambient temperature, *R* is the electrical resistance of the thermocouple, and *v*_*i*_, *ρ*_*i*_, and *H*_*i*_ are the volume, density, and volume-specific heat of the *i*th film material, respectively. The response time constant is usually obtained by τ = 1/(4*f*_*-3dB*_)^52^, where *f*_*-3dB*_ is the −3 dB cutoff frequency for the characteristics of the output voltage response and the infrared radiation frequency.

### TEDI film design

According to performance parameters (1)–(4) of the thermopile, the output voltage, responsivity, and detectivity are proportional to the thermocouple number, Seebeck coefficient difference, and infrared absorptivity and inversely proportional to the thermal conductance. In addition, the output voltage and detectivity are also proportional to the infrared absorber area. At present, the thermocouple number and Seebeck coefficient difference have been extensively optimized. Enhancing the performance of the thermopile by intrinsically improving the infrared absorption *η* and reducing the thermal conductance *G*_FL_ of the FLDI film should be a potential method to promote the development of high-performance thermopiles.

**Fig. 2:**
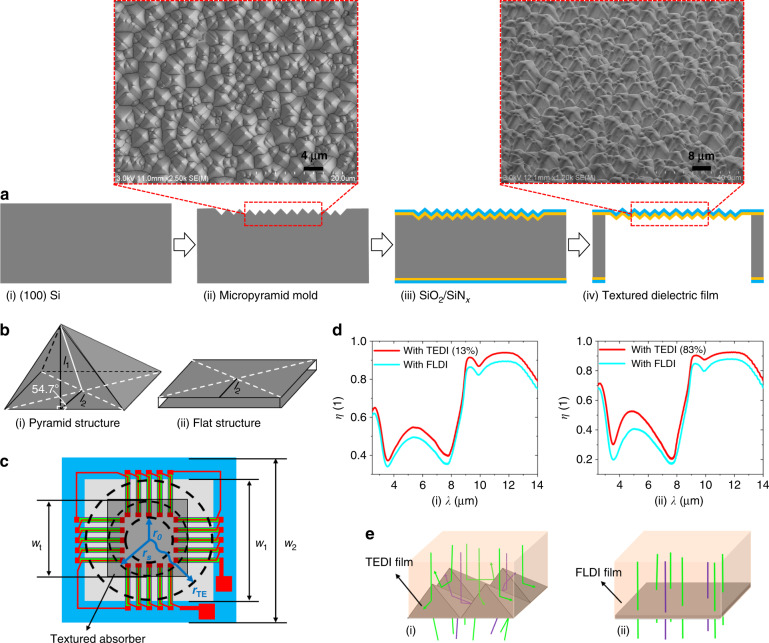
Fabrication and characterization of the TEDI film. **a** Hard-molding technology, **b** heat conduction paths *l*_1_ and *l*_2_ on the TEDI film and the FLDI film, **c** top view of the designed TEDI film-based thermopile, **d** infrared absorption of the infrared absorber with FLDI, 13% TEDI, and 83% TEDI, and **e** light paths on the surface of the TEDI film and the FLDI film.

Inspired by the characteristics of multiple reflection absorption of light and equivalent heat conduction extension of the random micropyramid black silicon material^53,54^, a novel pyramidal TEDI film is first designed and introduced into the typical thermopile, as shown in Fig. 1c and d. This TEDI film can be achieved by simple hard-molding technology, which uses random micropyramid silicon as a mold, as shown in Fig. 2a. The main processing steps are as follows.

(i) A (100) single-crystalline silicon wafer was used as a silicon substrate.

(ii) A random micropyramid silicon mold was made by a wet-etching process in a solution composed of 2 wt. % KOH, 5 vol. % isopropyl alcohol (IPA), and deionized (DI) water.

(iii) Both SiO_2_ and SiN_x_ dielectric films were formed on the surface of the random micropyramid silicon mold by high-temperature thermal oxidation and low-pressure chemical vapor deposition (LPCVD).

(iv) The front side of the silicon substrate was protected using photoresist, and then, a suspended TEDI film was achieved by backside dry etching.

The surface of the micropyramid silicon was composed of (111) crystal planes, as shown in Fig. 2b. An angle of 54.7° was formed between the (111) plane and the (100) bottom surface, which resulted from the anisotropic characteristics of the (100) silicon in the KOH solution^55^. Geometric analysis showed that path *l*_1_ of the pyramid structure was ~1/cos 54.7° ≈1.7 times that of the flat structure *l*_2_, as shown in Fig. 2b. Since the TEDI film was achieved by using micropyramid silicon as the mold in the hard-molding technology, the heat conduction path of the TEDI film should also be ~1.7 times that of the FLDI film.

Thus, the heat conduction path *r*_TE_ of a TEDI film-based infrared absorber (Fig. 2c) can be expressed as:

6 $$r_{{\mathrm{TE}}} = (r_{{\mathrm{FL}}} - r_s) + 1.7r_s$$

where *r*_*s*_ is the radius of the TEDI film region. Correspondingly, the thermal conductance *G*_1TE_ of the TEDI film-based infrared absorber can be expressed as:

7 $$G_{1{\mathrm{TE}}} = \mathop {\sum}\limits_j {\frac{{2\pi \lambda _jt_j}}{{\ln (r_{{\mathrm{TE}}}/r_0)}}}$$

Compared with the FLDI film-based infrared absorber (Fig. 1a), the increment Δ*G* in the structure thermal conductance of the TEDI film-based infrared absorber can be calculated by:

8 $${\Delta}G = \frac{{G_{1{\mathrm{TE}}} - G_{1{\mathrm{FL}}}}}{{G_{1{\mathrm{FL}}}}} = \frac{{\ln (r_{{\mathrm{FL}}}/r_0)}}{{\ln (r_{{\mathrm{TE}}}/r_0)}} - 1$$

Because *r*_TE_ is greater than *r*_FL_ and the ln(x) function is an increasing function, Δ*G* should be a negative value. In other words, the designed pyramidal TEDI film can reduce the thermal conductance of the conventional FLDI film. Moreover, the larger the textured area in the infrared absorber is, the higher the | Δ*G* | that can be achieved.

The infrared absorption of the achieved TEDI film-based infrared absorbers with different textured areas was tested by Fourier transform infrared spectroscopy (FT-IR), as shown in Fig. 2d. The textured size *w*_*t*_ of the TEDI film-based infrared absorbers (Fig. 2c) was 0.4 mm and 1.0 mm. The suspended size *w*_*1*_ and side length *w*_*2*_ of the test samples were 1.1 mm and 1.7 mm. Thus, the ratio between the textured area and the suspended area was ~13% and 83%, respectively. According to the infrared absorption spectrum (Fig. 2d), compared with the control FLDI film-based infrared absorber, the infrared absorption increment Δ*η* of the infrared absorber with 13% TEDI film and 83% TEDI film was ~7% and 13% in the range of 2.5–14 μm, respectively. This absorption enhancement may benefit from the multiple reflection absorption of the light incident on the surface of the TEDI film, as shown in Fig. 2e.

### TEDI film-based thermopile design

The output voltage response, responsivity, and detectivity of the thermopile are proportional to the infrared absorptivity *η* and inversely proportional to the thermal conductance *G* based on the infrared–thermal–electric conversion (1)–(4). Therefore, by introducing the above TEDI film-based infrared absorber into a conventional FLDI film-based thermopile, the performance improvement of the TEDI film-based thermopile can be estimated by:

9 $${\Delta}{\mathrm{performance}} = \frac{{1 + {\Delta}\eta }}{{1{\mathrm{ + }}{\Delta}G}} - 1$$

Equation (9) is mainly related to the infrared absorptivity *η* and the thermal conductance *G* of the infrared absorber. Referring to (8), the thermal conductance increment Δ*G* of the TEDI film-based infrared absorbers with 13% and 83% textured areas is approximately −18% and −30%, respectively. Thus, combined with their corresponding infrared absorption increments Δ*η* of 7% and 13%, the Δperformance of the TEDI film-based thermopiles with 13% and 83% textured areas can be up to ~30% and 61%, respectively.

**Fig. 3:**
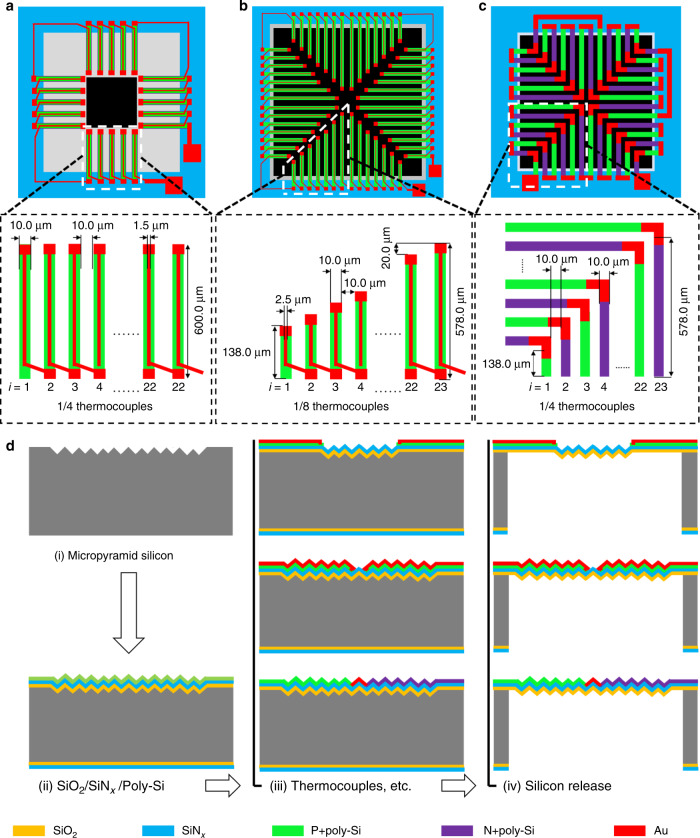
Schematic of the designed TEDI film-based thermopiles and their fabrication process. **a** Design_1-TEDI, **b** Design_2-TEDI, **c** Design_3-TEDI, and **d** fabrication process.

Figure 3a–c and 1d show a schematic and a cross-section of the designed TEDI film-based thermopiles. Design_1-TEDI with 88 P + poly-Si/Au thermocouples and a 13% textured area is first used to verify the theoretical performance improvement of ~30% estimated by (9). Then, Design_2-TEDI with 184 P + poly-Si/Au thermocouples and an 83% textured area is designed to verify the theoretical value of 61%. Simultaneously, this design can demonstrate the feasibility of patterning thermocouples on the surface of the textured infrared absorber to develop high-performance thermopiles. Finally, Design_3-TEDI with an 83% textured area is the optimization of Design_2-TEDI by replacing the 184 P + poly-Si/Au thermocouples with 92 P + /N + poly-Si thermocouples for greater detectivity.

P + poly-Si and N + poly-Si (thickness: 0.6 μm) were obtained by heavily doping boron and phosphorus ions into the poly-Si film with doses of 9 × 10^15^ cm^−2^ and 8 × 10^15^ cm^−2^ under the same energy of 90 keV, respectively. The thickness of the Au film is 0.4 μm. The tested resistivities of the formed P + poly-Si, N + poly-Si, and Au films obtained by a resistivity tester are ~40 Ω·μm, 15 Ω·μm, and 0.02 Ω·μm, respectively. Referring to other works^19,20^, the other properties of the three used materials are shown in Table 1. According to these material parameters, compared to Design_2-TEDI with 184 P + poly-Si/Au thermocouples, Design_3-TEDI with 92 P + /N + poly-Si thermocouples has the characteristics of half the number of thermocouples, approximately twice the Seebeck coefficient difference, a lower thermal conductance, and a smaller electrical resistance (~31% reduction).

**Table 1:** Properties of the three used materials.

Materials	Seebeck coefficient *α* (μV·K^−1^)	Thermal conductivity *λ* (W·m^-1^·K^−1^)
Au	1.9–2	310–320
P + poly-Si	150–160	28–30
N + poly-Si	−130 to −120	30–32

Referring to Eq. (4), the detectivity of the thermopile is proportional to the thermocouple number and Seebeck coefficient difference. Thus, the approximately twofold Seebeck coefficient difference of the P + /N + poly-Si thermocouple in Design_3-TEDI can be complementary to the performance effect caused by the decrease in the thermocouple number. On the other hand, the detectivity is also inversely proportional to the square root of the thermocouple resistance and the thermal conductance. Then, compared to Design_2-TEDI with 184 P + poly-Si/Au thermocouples, the detectivity enhancement of Design_3-TEDI with 92 P + /N + poly-Si thermocouples can be estimated by Eq. (10) ignoring the resistivity of Au.

10 $${\Delta}D^\ast = \left(\sqrt {\frac{{2\rho _{P + }}}{{\rho _{P + } + \rho _{N + }}}} - 1\right) + {\Delta}d$$

where *ρ*_*P+*_ and *ρ*_*N+*_ are the resistivities of the P + poly-Si and N + poly-Si films, respectively. The improvement in the detectivity of Design_3-TEDI can benefit from the reduction in the electrical resistance (1st item) and thermal conductance (2nd item) of the thermocouple. Although the thermal conductivity of the Au film is much higher than that of the P + /N + poly-Si films, the total thermal conductance of the 184 P + poly-Si/Au thermocouples in Design_2-TEDI is slightly larger (Δ*d*) than that of the 92 P + /N + poly-Si thermocouples in Design_3-TEDI, which is mainly due to the tiny width/length ratio of the Au leg. Referring to (10), the detectivity of Design_3-TEDI can be further improved by ~21% + Δ*d* compared to that of Design_2-TEDI.

Figure 3d shows the fabrication process of the TEDI film-based thermopiles. The main processing steps include (i) making a random micropyramid silicon mold, (ii) casting by thermal oxidation and LPCVD of SiN_x_, (iii) forming thermocouples, pads, and interconnects, and (iv) demolding by dry etching on the backside of the silicon substrate. The detailed process is as follows.

(i) The process started from an n-type (100) silicon wafer with 100 nm SiO_2_. A square window (side length: *w*_*t*_) open along the <110> orientation was formed by reactive ion etching (RIE) of SiO_2_. Then, a random micropyramid silicon mold was obtained in a solution composed of 2 wt. % KOH, 5 vol % IPA, and DI.

(ii) The TEDI film was formed on the surface of the random micropyramid silicon mold by the growth of 0.35 μm SiO_2_ and LPCVD of 1.0 μm SiN_x_.

(iii) The P + poly-Si and N + poly-Si thermocouple legs were patterned. A layer of 0.6 μm poly-Si film was first deposited on the substrate surface and then sequentially heavily doped with boron and phosphorus ions with doses of 9 × 10^15^ cm^−2^ and 8 × 10^15^ cm^−2^ under the same energy of 90 keV. Next, these thermocouple legs were patterned by deep reactive ion etching (DRIE) and passivated by thermal oxidation. Then, a layer of 30 nm/400 nm TiW/Au was deposited and etched to form the Au thermocouple legs, pads, and interconnects.

(iv) The front side of the silicon substrate was first protected with a thick LC100A photoresist, and then, the infrared absorber with the TEDI film was released by backside dry etching.

## Results and discussion

### Fabrication results of the designed thermopiles

**Fig. 4:**
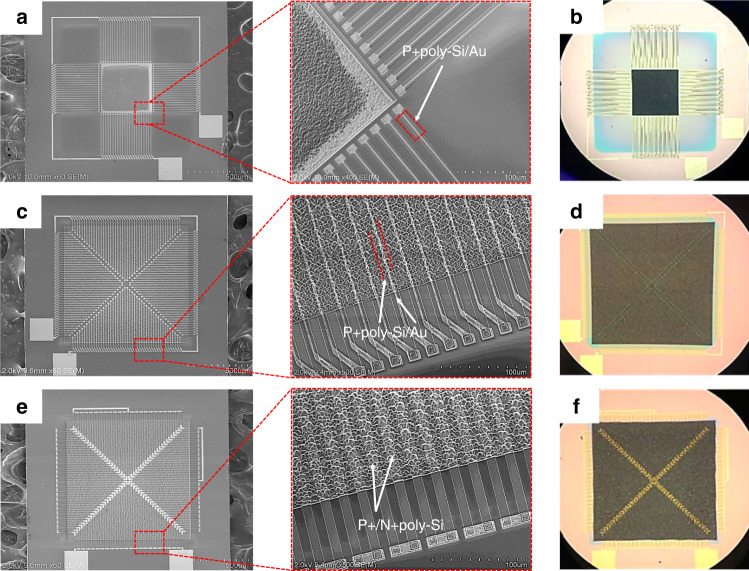
SEM images and optical photographs of the fabricated TEDI film-based thermopiles. **a**, **b** Design_1-TEDI, **c**, **d** Design_2-TEDI, and **e**, **f** Design_3-TEDI.

**Fig. 5:**
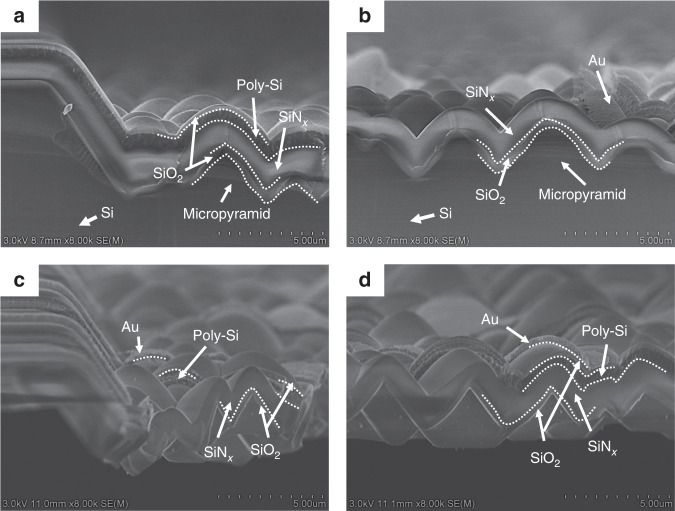
Cross-sectional SEM images of the formed TEDI film with a P + poly-Si/Au thermocouple. **a**, **b** Before and **c**, **d** after the release process.

Figure 4 shows SEM images and optical photographs of the achieved TEDI film-based thermopiles. To demonstrate the performance enhancement by using the TEDI film as the infrared absorber, a control FLDI film-based thermopile with the same thermocouple was simultaneously designed and fabricated (not shown). The SEM images show that all thermocouple legs were successfully patterned on the surface of the textured dielectric film. The optical photographs show that the formed TEDI film area in the infrared absorber of the TEDI film-based thermopiles is obviously black. This indicates that the TEDI film can be a highly antireflective film. Figure 5 shows cross-sectional SEM images of the textured SiO_2_/SiN_x_ dielectric film with a P + poly-Si/Au thermocouple. Before the release of the silicon substrate, the dielectric film was well attached to the surface of the micropyramid silicon mold. The top of the dielectric film became blunt. After release, the suspended dielectric film held the shape of the micropyramid structure.

### Test results of the fabricated thermopiles

Referring to Eqs. (1)–(5), to show the responsivity, detectivity, and response time constant of a thermopile, the electrical resistance, output voltage, and −3 dB cutoff frequency must first be obtained. The electrical resistance of the thermopile packaged with filter-free TO-5 was tested by a digital multimeter. The output voltage response and the response time constant were obtained by an IR testing system, which was composed of a blackbody source, a chopper, an amplifier circuit, and an oscilloscope. The infrared light emitted by the 500 K blackbody source was chopped at 4 Hz and then radiated onto the thermopile surface at a power density of 9 W·m^−2^. The output voltage of the thermopile was amplified 1240 times. At the same time, the 20 log- (*V*_out_/*V*_4 Hz_) frequency characteristics of the thermopile were tested by adjusting the chopper frequency from 4 to 40 Hz. The frequency at which the output voltage decays to −3 dB was used to calculate the response time constant. All test and calculation results were the average of six randomly selected samples for each thermopile of the different designs.

**Fig. 6:**
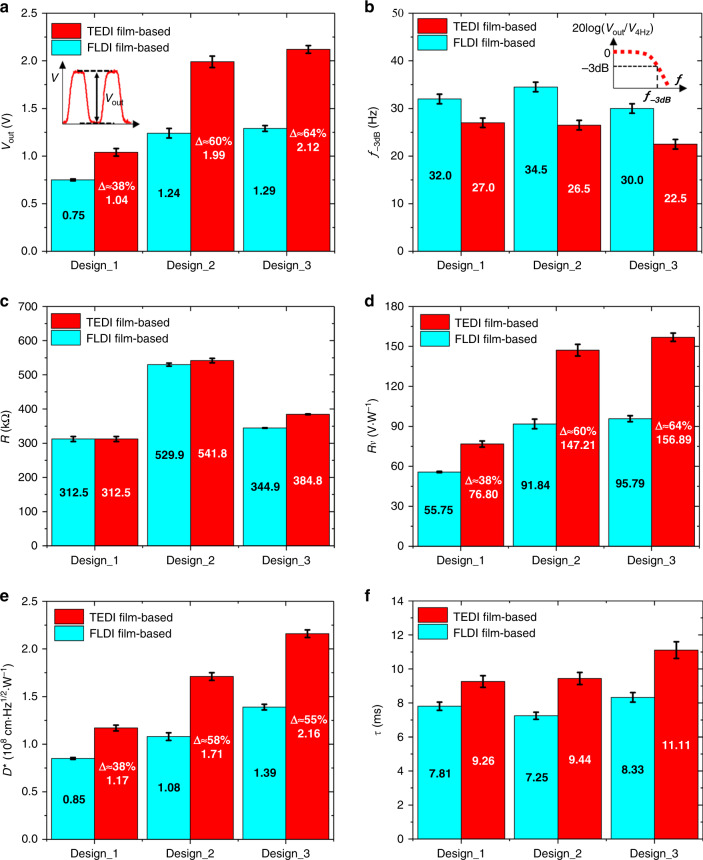
Performance of the fabricated thermopiles. Average **a** output voltage *V*_*out*_, **b** electrical resistance *R*, **c** −3 dB cutoff frequency *f*_*-3dB*_, **d** responsivity *Rv*, **e** detectivity D*, and **f** response time constant *τ* of the six randomly selected devices for each thermopile of the different designs.

The tested output voltage response, −3 dB cutoff frequency, and electrical resistance of the fabricated thermopiles are shown in Fig. 6a–c. Compared to the control FLDI film-based thermopiles, the tested output voltages of the TEDI film-based thermopiles are significantly enhanced by ~38%, 60%, and 64%, as shown in Fig. 6a. The output voltage of Design_3-TEDI is ~6.5% higher than that of Design_2-TEDI. In addition, the −3 dB cutoff frequencies of these TEDI film-based thermopiles are reduced, as shown in Fig. 6b. The reduction of Design_3-TEDI is close to that of Design_2-TEDI and higher than that of Design_1-TEDI. Finally, the electrical resistances of Design_2-TEDI and Design_3-TEDI are slightly increased, as shown in Fig. 6c. From Design_2 to Design_3, the electrical resistances of the FLDI film-based and TEDI film-based thermopiles are reduced by ~35% and 29%, respectively. These reductions are similar to the theoretical result (31%).

The responsivity, detectivity, and response time constant were calculated by Eqs. (3)–(5), and their results are shown in Fig. 6d–f. The responsivity and detectivity of the TEDI film-based thermopiles are significantly enhanced compared with those of the control FLDI film-based thermopiles. First, the increments in the responsivity of the TEDI film-based thermopiles are ~38%, 60%, and 64%, as shown in Fig. 6d. These results are equal to the increments in their output voltages. The responsivity (156.89 V·W^−1^) of Design_3-TEDI is the highest among the fabricated thermopiles. Second, the detectivity enhancements of the TEDI film-based thermopiles reach ~38%, 58%, and 55%, as shown in Fig. 6e. The last two increments are slightly lower than that in the corresponding output voltage response, while the first is the same. Design_3-TEDI also achieves the highest detectivity of 2.16 × 10^8^ cm·Hz^1/2^·W^−1^ among the fabricated thermopiles. Finally, the response time constants of the TEDI film-based thermopiles are slightly increased among the three designs, but all these are less than 12 ms, as shown in Fig. 6f.

## Discussion

Compared with the theoretical performance improvements of 30% and 61%, calculated by Eqs. (6)–(9), the experimental enhancements in the output voltage, responsivity, and detectivity in Design_1-TEDI and Design_2-TEDI are in good agreement with these values. With the same textured area, the enhancements in Design_2-TEDI and Design_3-TEDI are almost the same. Thus, compared with the control FLDI-based thermopile, the performance optimization of the designed TEDI film-based thermopile should mainly benefit from the enhancement in the infrared absorption of the TEDI film as well as the reduction in the thermal conductance. Obviously, the larger the textured area in the infrared absorber is, the better the device performance and the higher the improvement that can be achieved.

According to Eqs. (3) and (4), the responsivity and detectivity of a thermopile are proportional to the output voltage, while the detectivity is inversely proportional to the square root of the electrical resistance. When the thermocouple legs are patterned on the surface of the TEDI film, the resistance increases slightly due to the equivalent extension of these thermocouple legs, such as in Design_2-TEDI and Design_3-TEDI. Therefore, the enhancement in the responsivity of the TEDI film-based thermopile should be equal to the increment in the output voltage. Correspondingly, the detectivity improvements of Design_2-TEDI and Design_3-TEDI should be slightly lower than the increments in their output voltages. Referring to Eq. (5), since the response time constant is inversely proportional to the thermal conductance of the TEDI film absorber, the response time constant of the TEDI film-based thermopile increases, and its −3 dB cutoff frequency is reduced.

Table 2 shows the performance parameters of cost-effective thermopiles. Compared with other thermopiles, Design_2-TEDI and Design_3-TEDI achieve an excellent performance combination. Their responsivity and detectivity can reach more than 140 V·W^−1^ and 1.7 × 10^8^ cm·Hz^1/2^·W^−1^, respectively. Importantly, the detectivity (2.16 × 10^8^ cm·Hz^1/2^·W^−1^) of Design_3-TEDI exhibits the best in-air performance reported to date for a series of cost-effective thermopiles. Furthermore, the detectivity of Design_3-TEDI is ~26% higher than that of Design_2-TEDI, which is consistent with the theoretical value (21%+Δ*d*) calculated by Eq. (10). Δ*d* is the voltage increment (6.5%) of the TEDI film-based thermopile Design_3-TEDI compared to Design_2-TEDI. Due to the inverse relationship between the output voltage and thermal conductance, Δ*d* can be regarded as the reduction in the thermal conductance of the P + /N + poly-Si thermocouples.

**Table 2:** Performances of cost-effective thermopiles in air.

Years	Materials	*Rv* (V·W^−1^)	D* (10^8^ cm·Hz^1/2^·W^−1^)	*τ* (ms)
1996	P + Si/Au (F)^56^	121	0.95	23
2005	N + poly-Si/Al (F)^13^	114.5	1.07	/
2007	N + poly-Si/Al (F)^15^	63	1.23	/
2010	P + poly-Si/Al (C)^25^	43.5	0.25	14.1
2010	N + poly-Si/Al (C)^57^	102	0.92	16.8
2010	P + poly-Si/Al (F)^58^	31.65	1.16	/
2011	N + poly-Si/Ti (F)^16^	62.8	1.88	17
2013	P + /N + poly-Si (C)^59^	88.5	1.24	/
2015	P + /N + poly-Si (C)^18^	425.7	0.13	33
2015	N + poly-Si/Al (C)^60^	22.2	0.89	1.27
2018	P + /N + poly-Si (C)^61^	160.3	0.98	2.5
2019	P + Si/Al (C)^20^	342	0.56	0.56
Our works	P + poly-Si/Au (F)	91.84	1.08	7.25
	P + poly-Si/Au (T)	147.21	1.71	9.44
	P + /N + poly-Si (F)	95.79	1.39	8.33
	P + /N + poly-Si (T)	156.89	2.16	11.11

*F* FLDI film-based, *T* TEDI film-based, *C* Cantilever beam-based.

## Conclusion

A new performance-enhancement strategy for thermopiles by simultaneously controlling their heat conduction and infrared absorption with a TEDI film is developed and presented in this paper. Control via the TEDI film of the heat conduction and infrared absorption as well as performance enhancement of the TEDI film-based thermopile are systematically analyzed and demonstrated. Compared to the control FLDI film, the thermal conductance of the TEDI film can be reduced by ~18– 30%, while the infrared absorption can be increased by ~7–13%. Correspondingly, the responsivity and detectivity of the fabricated TEDI film-based thermopile can be significantly enhanced by ~38–64%, which is in good agreement with the theoretical result (30–61%). An optimized TEDI film-based thermopile has achieved a responsivity of 156.89 V·W^−1^ and a detectivity of 2.16 × 10^8^ cm·Hz^1/2^·W^−1^, while the time constant is still <12 ms. Moreover, the detectivity of the optimized thermopile has exhibited the best in-air performance reported to date for a series of cost-effective thermopiles. These results demonstrate the feasibility of using this new strategy to simultaneously control the heat conduction and infrared absorption to achieve high-performance thermopiles and other sensors with heat transfer and/or infrared absorption mechanisms.

## References

[1] Xu DH, Wang YL, Xiong B, Li T (2017). MEMS-based thermoelectric infrared sensors: a review. Front. Mech. Eng.-Prc..

[2] Xu DH, Xiong B, Wang YL, Li T (2011). Robust array-composite micromachined thermopile IR detector by CMOS technology. IEEE Electr. Device Lett..

[3] Popa D, Ali SZ, Hoppe R, Dai Y, Udrea F (2019). Smart CMOS mid-infrared sensor array. Opt. Lett..

[4] Gu NH, Yang B, Zhang T (2020). Dynamic fuzzy background removal for indoor human target perception based on thermopile array sensor. IEEE Sens. J..

[5] Vincent TA, Gardner JW (2016). A low cost MEMS based NDIR system for the monitoring of carbon dioxide in breath analysis at ppm levels. Sens. Actuat. B Chem..

[6] de Hoyos-Vazquez FF, Carreno-de Leon MC, Serrano-Nunez EO, Flores-Alamo N, Rios MJS (2019). Development of a novel non-dispersive infrared multi sensor for measurement of gases in sediments. Sens. Actuat. B Chem..

[7] Yan JB, Liao XP, Yan DY, Chen YG (2018). Review of micro thermoelectric generator. J. Microelectromech. Syst..

[8] Deng F, Qiu HB, Chen J, Wang L, Wang B (2017). Wearable thermoelectric power generators combined with flexible supercapacitor for low-power human diagnosis devices. IEEE Trans. Ind. Electron.

[9] Ke WJ, Liu M, Li T, Wang YL (2019). MEMS thermal gas flow sensor with self-test function. J. Micromech. Microeng..

[10] Tian W, Wang Y, Zhou H, Wang YL, Li T (2020). Micromachined thermopile based high heat flux sensor. J. Microelectromech. Syst..

[11] Choi IH, Wise KD (1986). A silicon-thermopile-based infrared sensing array for use in automated manufacturing. IEEE Trans. Electron Dev..

[12] Socher E, Bochobza-Degani O, Nemirovsky Y (2000). Optimal performance of CMOS compatible IR thermoelectric sensors. J. Microelectromech. Syst..

[13] Hartwig, S. et al. A highly sensitive IR-optical sensor for ethylene-monitoring. in SPIE Proceedings Vol. 5836: Smart Sensors, Actuators, and MEMS II (eds Cane, C., Chiao, J.-C., & Verdu, F. V.), 452–460 (International Society for Optics and Photonics, 2005).

[14] Chen CN, Huang WC (2011). A CMOS-MEMS thermopile with low thermal conductance and a near-perfect emissivity in the 8-14-mu m wavelength range. IEEE Electr. Device Lett..

[15] Chen SJ, Shen CH (2007). A new high-filling-factor CMOS-compatible thermopile. IEEE Trans. Instrum. Meas..

[16] Chen CN (2011). Temperature error analysis and parameter extraction of an 8-14-mu m thermopile with a wavelength-independent absorber for tympanic thermometer. IEEE Sens. J..

[17] Allison SC, Smith RL, Howard DW, Gonzalez C, Collins SD (2003). A bulk micromachined silicon thermopile with high sensitivity. Sens. Actuat. A Phys..

[18] Zhou HC, Kropelnicki P, Lee CK (2015). CMOS compatible midinfrared wavelength-selective thermopile for high temperature applications. J. Microelectromech. Syst..

[19] Xie J, Lee C, Wang MF, Liu YH, Feng HH (2009). Characterization of heavily doped polysilicon films for CMOS-MEMS thermoelectric power generators. J. Micromech. Microeng..

[20] Li W, Ni Z, Wang JC, Li XX (2019). A front-side microfabricated tiny-size thermopile infrared detector with high sensitivity and fast response. IEEE Trans. Electron Dev..

[21] Schieferdecker J, Quad R, Holzenkampfer E, Schulze M (1995). Infrared thermopile sensors with high-sensitivity and very-low temperature-coefficient. Sens. Actuat a-Phys..

[22] Chen CN (2012). Fully quantitative characterization of CMOS-MEMS polysilicon/titanium thermopile infrared sensors. Sens. Actuat B-Chem..

[23] Xu W, Wang XY, Zhao X, Lee YK (2020). Two-dimensional CMOS MEMS thermal flow sensor with high sensitivity and improved accuracy. J. Microelectromech. Syst..

[24] Yu HT, Xu PC, Xia XY, Lee DW, Li XX (2012). Micro-/nanocombined gas sensors with functionalized mesoporous thin film self-assembled in batches onto resonant cantilevers. IEEE Trans. Ind. Electron.

[25] Xu DH, Xiong B, Wang YL (2010). Self-aligned thermoelectric infrared sensors with post-CMOS micromachining. IEEE Electr. Device Lett..

[26] Simon T, Barsan N, Bauer M, Weimar U (2001). Micromachined metal oxide gas sensors: opportunities to improve sensor performance. Sens. Actuat. B Chem..

[27] Xie DC (2019). A low power cantilever-based metal oxide semiconductor gas sensor. IEEE Electr. Device Lett..

[28] Qiu L (2018). Advances in thermal transport properties at nanoscale in China. Int. J. Heat. Mass. Tran..

[29] Qiu L (2020). A review of recent advances in thermophysical properties at the nanoscale: from solid state to colloids. Phys. Rep..

[30] Qiu L (2018). Inhomogeneity in pore size appreciably lowering thermal conductivity for porous thermal insulators. Appl. Therm. Eng..

[31] Qiu L (2015). Adaptable thermal conductivity characterization of microporous membranes based on freestanding sensor-based 3 omega technique. Int. J. Therm. Sci..

[32] Xu DH, Xiong B, Wang YL (2011). Micromachined thermopile IR detector module with high performance. Ieee Photonic Tech. Lett..

[33] Kischkat J (2012). Mid-infrared optical properties of thin films of aluminum oxide, titanium dioxide, silicon dioxide, aluminum nitride, and silicon nitride. Appl. Opt..

[34] Ashraf S, Mattsson CG, Thungstrom G (2019). Fabrication and characterization of a SU-8 epoxy membrane-based thermopile detector with an integrated multilayered absorber structure for the Mid-IR region. IEEE Sens J..

[35] De Luca A (2015). Enhanced spectroscopic gas sensors using in-situ grown carbon nanotubes. Appl Phys. Lett..

[36] Li H (2020). Self-assembly of carbon Black/AAO templates on nanoporous Si for broadband infrared absorption. ACS Appl. Mater. Inter.

[37] Zhang MY, Ban DY, Xu C, Yeow JTW (2019). Large-area and broadband thermoelectric infrared detection in a carbon nanotube black-body absorber. ACS Nano.

[38] Panjwani D (2014). Stencil lithography of gold-black IR absorption coatings. Infrared Phys. Techn..

[39] Smith EM (2016). Dual band sensitivity enhancements of a VOx microbolometer array using a patterned gold black absorber. Appl Opt..

[40] Li CH (2016). Fabrication of black silicon with thermostable infrared absorption by femtosecond laser. IEEE Photonics J..

[41] Yu XY, Zhao JH, Li CH, Chen QD, Sun HB (2017). Gold-hyperdoped black silicon with high IR absorption by femtosecond laser irradiation. IEEE Trans. Nanotechnol..

[42] Hao JM (2010). High performance optical absorber based on a plasmonic metamaterial. Appl. Phys. Lett..

[43] Ogawa S, Kimata M (2017). Wavelength- or polarization-selective thermal infrared detectors for multi-color or polarimetric imaging using plasmonics and metamaterials. Materials.

[44] Ogawa S, Komoda J, Masuda K, Kimata M (2013). Wavelength selective wideband uncooled infrared sensor using a two-dimensional plasmonic absorber. Opt. Eng..

[45] Ogawa S, Okada K, Fukushima N, Kimata M (2012). Wavelength selective uncooled infrared sensor by plasmonics. Appl. Phys. Lett..

[46] Ogawa S, Masuda K, Takagawa Y, Kimata M (2014). Polarization-selective uncooled infrared sensor with asymmetric two-dimensional plasmonic absorber. Opt. Eng..

[47] Lin PS, Shen TW, Chan KC, Fang W (2020). CMOS MEMS thermoelectric infrared sensor with plasmonic metamaterial absorber for selective wavelength absorption and responsivity enhancement. IEEE Sens J..

[48] Ogawa S, Takagawa Y, Kimata M (2018). Broadband polarization-selective uncooled infrared sensors using tapered plasmonic micrograting absorbers. Sens. Actuat. A Phys..

[49] Shen QC (2018). Bioinspired infrared sensing materials and systems. Adv. Mater..

[50] He YQ, Wang YL, Li T (2020). Performance enhanced thermopile with rough dielectric film black. IEEE Electr. Device Lett..

[51] He YQ, Wang YL, Li T (2020). Improved thermopile on pyramidally-textured dielectric film. IEEE Electr. Device L.

[52] Graf A, Arndt M, Sauer M, Gerlach G (2007). Review of micromachined thermopiles for infrared detection. Meas. Sci. Technol..

[53] Wooh S (2013). Efficient light harvesting with micropatterned 3D pyramidal photoanodes in dye-sensitized solar cells. Adv. Mater..

[54] Zhong SH, Wang WJ, Tan M, Zhuang YF, Shen WZ (2017). Realization of quasi-omnidirectional solar cells with superior electrical performance by all-solution-processed Si nanopyramids. Adv. Sci..

[55] Zhong SH, Wang WJ, Zhuang YF, Huang ZG, Shen WZ (2016). All-solution-processed random Si nanopyramids for excellent light trapping in ultrathin solar cells. Adv. Funct. Mater..

[56] Muller M (1996). A thermoelectric infrared radiation sensor with monolithically integrated amplifier stage and temperature sensor. Sens. Actuat. A Phys..

[57] Xu DH, Xiong B, Wang YL (2010). Design, fabrication and characterization of a front-etched micromachined thermopile for IR detection. J. Micromech. Microeng..

[58] Wang KQ (2010). Thermopile infrared detector with detectivity greater than 10(8) cmHz((1/2))/W. J. Infrared Millim. Te.

[59] Zhou HC, Kropelnicki P, Tsai JM, Lee C (2013). Development of a thermopile infrared sensor using stacked double polycrystalline silicon layers based on the CMOS process. J. Micromech. Microeng..

[60] Zhou HC, Kropelnicki P, Lee C (2015). Characterization of nanometer-thick polycrystalline silicon with phonon-boundary scattering enhanced thermoelectric properties and its application in infrared sensors. Nanoscale.

[61] Ke WJ, Wang Y, Zhou H, Li T, Wang YL (2018). Design, fabrication, and characterization of a high-performance CMOS-compatible thermopile infrared detector with self-test function. J. Micromech. Microeng..

